# 2,3-*O*-Isopropyl­idene-3-*C*-phenyl­erythrofuran­ose

**DOI:** 10.1107/S160053680904848X

**Published:** 2009-11-21

**Authors:** Tony V. Robinson, Dennis K. Taylor, Edward R. T. Tiekink

**Affiliations:** a Discipline of Chemistry, University of Adelaide, 5005 South Australia, Australia; b Discipline of Wine and Horticulture, University of Adelaide, Waite Campus, Glen, Osmond 5064, South Australia, Australia; c Department of Chemistry, University of Malaya, 50603 Kuala Lumpur, Malaysia

## Abstract

The title compound, C_13_H_16_O, comprises two fused five-membered rings. Each ring has an envelope conformation, with the ether O atom in the furan­ose ring, and the CMe_2_ atom in the acetonide ring as the flap atoms. In the crystal, centrosymmetrically related mol­ecules associate *via* hydr­oxy–ether O—H⋯O hydrogen bonds and the resulting dimers are linked into a supra­molecular chain with a flattened topology *via* C—H⋯O_hydr­oxy_ contacts, and aligned in the *a*-axis direction.

## Related literature

For the relevance and chemistry of systems related to the title compound, see: Pedersen *et al.* (2009 ▶); Robinson *et al.* (2006 ▶, 2009 ▶); Valente *et al.* (2009 ▶). For the reactions of Co(II) complexes with endoperoxides, see: Boyd *et al.* (1980 ▶); Sutbeyaz *et al.* (1988 ▶); Greatrex *et al.* (2003 ▶); Greatrex & Taylor (2005 ▶).
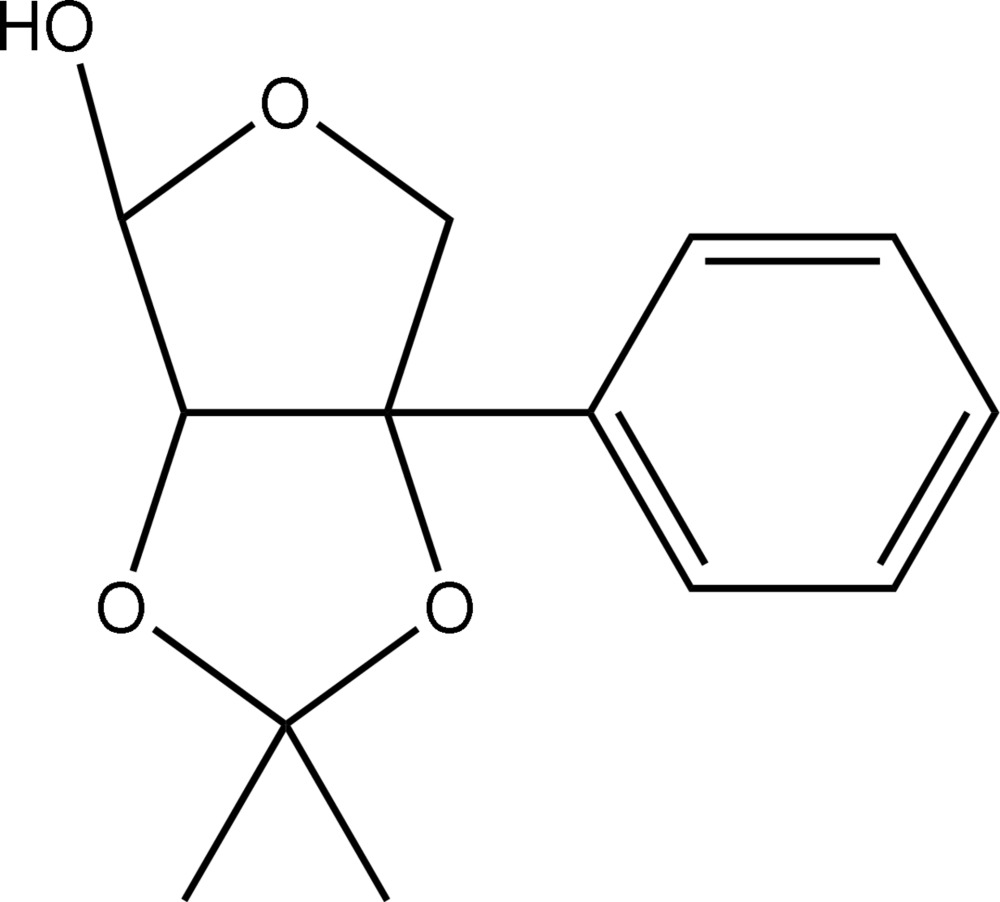


## Experimental

### 

#### Crystal data

C_13_H_16_O_4_
*M* *_r_* = 236.26
Triclinic, 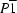

*a* = 5.716 (2) Å
*b* = 9.201 (4) Å
*c* = 11.871 (6) Å
α = 89.76 (3)°
β = 78.72 (2)°
γ = 73.70 (2)°
*V* = 586.9 (4) Å^3^
*Z* = 2
Mo *K*α radiation
μ = 0.10 mm^−1^
*T* = 173 K
0.35 × 0.35 × 0.10 mm

#### Data collection

Rigaku AFC12κ/SATURN724 diffractometer
Absorption correction: multi-scan (*ABSCOR*; Higashi, 1995 ▶) *T*_min_ = 0.773, *T*_max_ = 1.000
14572 measured reflections
2408 independent reflections
2361 reflections with *I* > 2σ(*I*)
*R*_int_ = 0.030

#### Refinement

*R*[*F* ^2^ > 2σ(*F* ^2^)] = 0.045
*wR*(*F* ^2^) = 0.157
*S* = 1.16
2408 reflections
157 parameters
1 restraint
H-atom parameters constrained
Δρ_max_ = 0.27 e Å^−3^
Δρ_min_ = −0.25 e Å^−3^

### 

Data collection: *CrystalClear* (Rigaku/MSC, 2005 ▶); cell refinement: *CrystalClear*; data reduction: *CrystalClear*; program(s) used to solve structure: *SHELXS97* (Sheldrick, 2008 ▶); program(s) used to refine structure: *SHELXL97* (Sheldrick, 2008 ▶); molecular graphics: *ORTEPII* (Johnson, 1976 ▶) and *DIAMOND* (Brandenburg, 2006 ▶); software used to prepare material for publication: *publCIF* (Westrip, 2009 ▶).

## Supplementary Material

Crystal structure: contains datablocks global, I. DOI: 10.1107/S160053680904848X/sj2687sup1.cif

Structure factors: contains datablocks I. DOI: 10.1107/S160053680904848X/sj2687Isup2.hkl

Additional supplementary materials:  crystallographic information; 3D view; checkCIF report

## Figures and Tables

**Table 1:** Hydrogen-bond geometry (Å, °)

*D*—H⋯*A*	*D*—H	H⋯*A*	*D*⋯*A*	*D*—H⋯*A*
O2—H2o⋯O1^i^	0.84	1.93	2.755 (2)	166
C5—H5a⋯O2^ii^	0.99	2.47	3.296 (3)	140

Symmetry codes: (i) 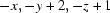; (ii) 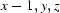.

**Fig. 1.**
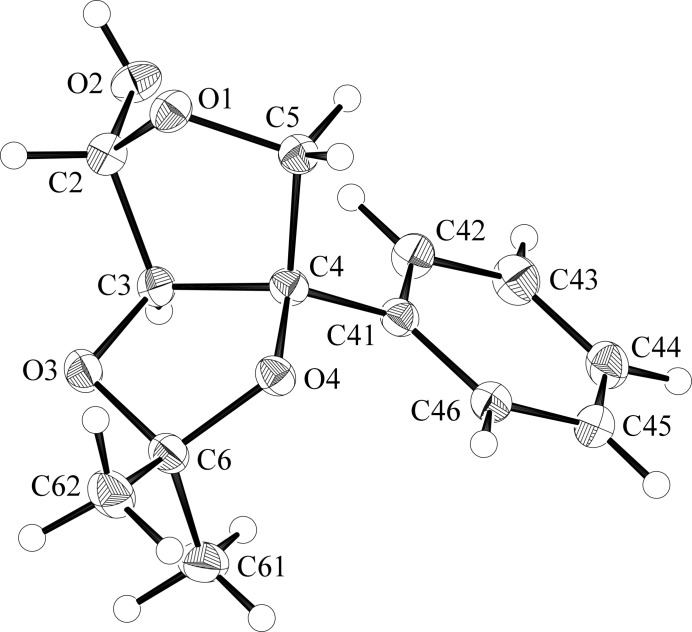
Molecular structure of (I) showing atom-labelling scheme and displacement ellipsoids at the 35% probability level.

**Fig. 2.**
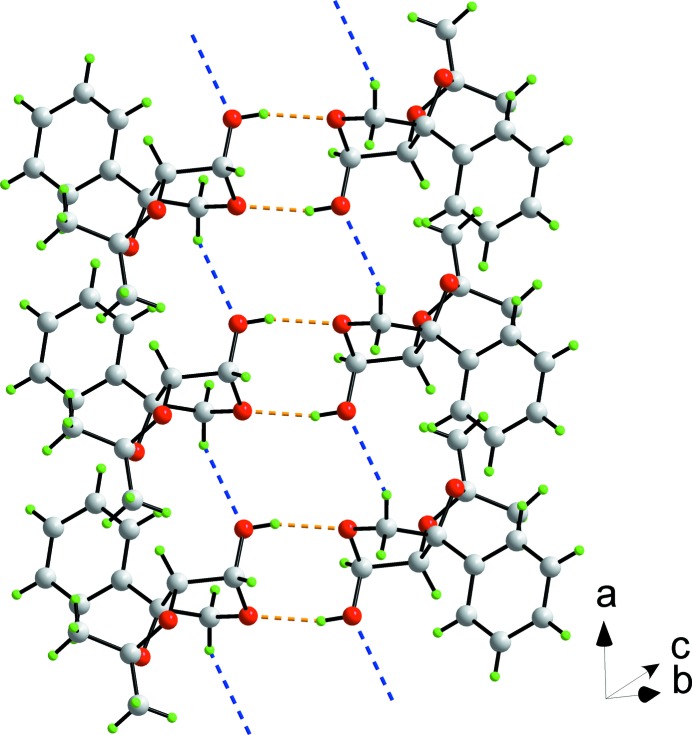
Supramolecular chain formation along the a axis in (I) mediated by O—H···O hydrogen bonds (orange dashed lines) and C—H···O contacts (blue dashed lines).

## supplementary crystallographic information

### Comment

The dihydroxyation of monocyclic and bicyclic 1,2-dioxines has provided a new route for the stereoselective synthesis of a diverse range of carbohydrates and related compounds (Pedersen *et al.*, 2009; Robinson *et al.*, 2006; Robinson *et al.*, 2009; Valente *et al.*, 2009). During the course of these studies, the title compound, (I), was obtained by the Co(II)-mediated ring-opening of the precursor 1,2-dioxane, post dihydroxyation. The reactions of Co(II) complexes with endoperoxides have been well documented (Boyd *et al.*, 1980; Sutbeyaz *et al.*, 1988; Greatrex *et al.*, 2003; Greatrex & Taylor, 2005).

The molecular structure of (I), Fig. 1, comprises two fused five-membered rings linked at the C3—C4 bond. Each of the five-membered rings adopts an envelope conformation, on atom O1 for the furanose (O1, C2—C5) ring, and on atom C6 for the acetonide (O3, O4, C3, C4, C6) ring. When viewed down the C3—C4 axis, the O1 atom lies above the plane through the four remaining atoms, away from the phenyl substituent and the C6 atom lies below the plane, being orientated in the same direction as the phenyl ring. In the crystal structure centrosymmetrically related pairs of molecules associate *via* O—H···O hydrogen bonds to form an eight-membered {···OCOH}_2_ synthon, Table 1 and Fig. 2. The dimers are linked into a supramolecular chain *via* C—H···O contacts and ten-membered {···OH···OCH}_2_ synthons, Table 1. The resulting chain comprising alternating eight- and ten-membered synthons has a flattened topology, Fig. 2, and is aligned along the *a* axis.

### Experimental

For full synthetic procedures and characterization data see Pedersen *et al.* (2009) and Robinson *et al.* (2009). To a stirred solution of Co(salen)_2_ (17 mg, 0.05 mmol) in THF (5 ml) at ambient temperature was added (*3aR*,*7aS*)-3a-phenyl-tetrahydro-2,2-dimethyl-[1,3]dioxolo[4,5-*d*][1,2]dioxine (501 mg, 2.12 mmol), and the reaction left to stir until complete by TLC (~16 h). All volatiles were removed *in vacuo* giving a crude mixture of regioisomers in a 40:60 ratio. The isomers were fully separated by flash chromatography giving a combined total yield of 496 mg (99%). Compound (I) was isolated as a colourless solid (198 mg), and the pure material was recrystallized from a slowly evaporating 1:1 mixture of dichloromethane/heptane to give colourless prisms, m. pt. 424–425 K. The compound was found to exist solely in its cyclic hemi-acetal form(*s*) both as a solid indicated by IR (absence of carbonyl signal), and in CDCl_3_ solution which revealed a 90:10 ratio of anomers.

### Refinement

Carbon-bound H-atoms were placed in calculated positions (C–H 0.95–1.00 Å) and were included in the refinement in the riding model approximation with *U*_iso_(H) set to 1.2–1.5*U*_eq_(C). The O–bound H-atom was located in a difference Fourier map and was refined with an O–H restraint of 0.840±0.001 Å, and with *U_iso_*(H) = 1.*5U_eq_*(O).

### Crystal data

**Table:**

C_13_H_16_O_4_	*Z* = 2
*M**_r_* = 236.26	*F*(000) = 252
Triclinic, *P*1	*D*_x_ = 1.337 Mg m^−^^3^
Hall symbol: -P 1	Mo *K*α radiation, λ = 0.71070 Å
*a* = 5.716 (2) Å	Cell parameters from 2428 reflections
*b* = 9.201 (4) Å	θ = 3.5–27.5°
*c* = 11.871 (6) Å	µ = 0.10 mm^−^^1^
α = 89.76 (3)°	*T* = 173 K
β = 78.72 (2)°	Prism, pale-yellow
γ = 73.70 (2)°	0.35 × 0.35 × 0.10 mm
*V* = 586.9 (4) Å^3^	

### Data collection

**Table:**

Rigaku AFC12κ/SATURN724 diffractometer	2408 independent reflections
Radiation source: fine-focus sealed tube	2361 reflections with *I* > 2σ(*I*)
graphite	*R*_int_ = 0.030
ω scans	θ_max_ = 26.5°, θ_min_ = 1.8°
Absorption correction: multi-scan (*ABSCOR*; Higashi, 1995)	*h* = −7→7
*T*_min_ = 0.773, *T*_max_ = 1.000	*k* = −10→11
14572 measured reflections	*l* = −14→14

### Refinement

**Table:**

Refinement on *F*^2^	Primary atom site location: structure-invariant direct methods
Least-squares matrix: full	Secondary atom site location: difference Fourier map
*R*[*F*^2^ > 2σ(*F*^2^)] = 0.045	Hydrogen site location: inferred from neighbouring sites
*wR*(*F*^2^) = 0.157	H-atom parameters constrained
*S* = 1.16	*w* = 1/[σ^2^(*F*_o_^2^) + (0.0929*P*)^2^ + 0.1356*P*] where *P* = (*F*_o_^2^ + 2*F*_c_^2^)/3
2408 reflections	(Δ/σ)_max_ < 0.001
157 parameters	Δρ_max_ = 0.27 e Å^−^^3^
1 restraint	Δρ_min_ = −0.25 e Å^−^^3^

### Special details

**Table:**

Geometry. All e.s.d.'s (except the e.s.d. in the dihedral angle between two l.s. planes) are estimated using the full covariance matrix. The cell e.s.d.'s are taken into account individually in the estimation of e.s.d.'s in distances, angles and torsion angles; correlations between e.s.d.'s in cell parameters are only used when they are defined by crystal symmetry. An approximate (isotropic) treatment of cell e.s.d.'s is used for estimating e.s.d.'s involving l.s. planes.
Refinement. Refinement of *F*^2^ against ALL reflections. The weighted *R*-factor *wR* and goodness of fit *S* are based on *F*^2^, conventional *R*-factors *R* are based on *F*, with *F* set to zero for negative *F*^2^. The threshold expression of *F*^2^ > σ(*F*^2^) is used only for calculating *R*-factors(gt) *etc*. and is not relevant to the choice of reflections for refinement. *R*-factors based on *F*^2^ are statistically about twice as large as those based on *F*, and *R*- factors based on ALL data will be even larger.

### Fractional atomic coordinates and isotropic or equivalent isotropic displacement parameters (Å^2^)

**Table:**

	*x*	*y*	*z*	*U*_iso_*/*U*_eq_	
O1	−0.11375 (18)	0.89747 (11)	0.40885 (9)	0.0294 (3)	
O2	0.29860 (19)	0.86868 (13)	0.42473 (9)	0.0335 (3)	
H2O	0.2683	0.9394	0.4747	0.050*	
O3	0.1029 (2)	0.87393 (12)	0.15795 (9)	0.0321 (3)	
O4	−0.09610 (17)	0.69527 (11)	0.20274 (8)	0.0270 (3)	
C2	0.1242 (3)	0.91139 (17)	0.35425 (12)	0.0281 (3)	
H2	0.1102	1.0170	0.3296	0.034*	
C3	0.2058 (3)	0.79968 (16)	0.25019 (12)	0.0264 (3)	
H3	0.3905	0.7548	0.2291	0.032*	
C4	0.0642 (2)	0.67820 (16)	0.28391 (11)	0.0248 (3)	
C5	−0.0944 (3)	0.73843 (16)	0.40319 (12)	0.0273 (3)	
H5A	−0.2612	0.7226	0.4120	0.033*	
H5B	−0.0130	0.6864	0.4645	0.033*	
C6	−0.0013 (3)	0.77251 (17)	0.10732 (12)	0.0300 (4)	
C41	0.2235 (2)	0.51612 (16)	0.28472 (12)	0.0263 (3)	
C42	0.4230 (3)	0.48304 (18)	0.34152 (13)	0.0322 (4)	
H42	0.4620	0.5630	0.3772	0.039*	
C43	0.5645 (3)	0.33463 (19)	0.34623 (15)	0.0380 (4)	
H43	0.7000	0.3135	0.3849	0.046*	
C44	0.5092 (3)	0.21693 (18)	0.29478 (14)	0.0378 (4)	
H44	0.6059	0.1151	0.2982	0.045*	
C45	0.3115 (3)	0.24891 (18)	0.23827 (13)	0.0362 (4)	
H45	0.2735	0.1686	0.2025	0.043*	
C46	0.1683 (3)	0.39770 (17)	0.23361 (12)	0.0307 (4)	
H46	0.0323	0.4184	0.1953	0.037*	
C61	0.1984 (3)	0.6632 (2)	0.02004 (14)	0.0398 (4)	
H61A	0.1252	0.5942	−0.0141	0.060*	
H61B	0.3294	0.6045	0.0582	0.060*	
H61C	0.2701	0.7202	−0.0405	0.060*	
C62	−0.2175 (3)	0.8637 (2)	0.05769 (14)	0.0397 (4)	
H62A	−0.2898	0.7948	0.0229	0.060*	
H62B	−0.1591	0.9276	−0.0011	0.060*	
H62C	−0.3439	0.9279	0.1191	0.060*	

### Atomic displacement parameters (Å^2^)

**Table:**

	*U*^11^	*U*^22^	*U*^33^	*U*^12^	*U*^13^	*U*^23^
O1	0.0253 (5)	0.0272 (6)	0.0334 (6)	−0.0055 (4)	−0.0032 (4)	−0.0028 (4)
O2	0.0280 (6)	0.0361 (6)	0.0352 (6)	−0.0045 (4)	−0.0103 (4)	−0.0069 (4)
O3	0.0409 (6)	0.0306 (6)	0.0301 (6)	−0.0151 (5)	−0.0124 (4)	0.0082 (4)
O4	0.0261 (5)	0.0309 (6)	0.0258 (5)	−0.0093 (4)	−0.0082 (4)	0.0054 (4)
C2	0.0248 (7)	0.0279 (7)	0.0316 (7)	−0.0071 (5)	−0.0062 (5)	0.0009 (6)
C3	0.0258 (7)	0.0266 (7)	0.0272 (7)	−0.0083 (5)	−0.0051 (5)	0.0028 (5)
C4	0.0236 (7)	0.0282 (8)	0.0237 (7)	−0.0080 (5)	−0.0066 (5)	0.0025 (5)
C5	0.0258 (7)	0.0279 (8)	0.0274 (7)	−0.0071 (5)	−0.0042 (5)	0.0014 (5)
C6	0.0343 (8)	0.0326 (8)	0.0258 (7)	−0.0128 (6)	−0.0079 (6)	0.0056 (6)
C41	0.0265 (7)	0.0271 (8)	0.0243 (6)	−0.0075 (6)	−0.0030 (5)	0.0025 (5)
C42	0.0308 (8)	0.0308 (8)	0.0358 (8)	−0.0077 (6)	−0.0105 (6)	0.0022 (6)
C43	0.0332 (8)	0.0367 (9)	0.0420 (9)	−0.0038 (6)	−0.0114 (7)	0.0070 (7)
C44	0.0419 (9)	0.0275 (8)	0.0365 (8)	−0.0006 (6)	−0.0037 (7)	0.0053 (6)
C45	0.0479 (9)	0.0276 (8)	0.0325 (8)	−0.0108 (7)	−0.0065 (7)	0.0011 (6)
C46	0.0347 (8)	0.0304 (8)	0.0283 (7)	−0.0102 (6)	−0.0080 (6)	0.0031 (6)
C61	0.0430 (9)	0.0468 (10)	0.0281 (8)	−0.0141 (7)	−0.0015 (6)	−0.0018 (7)
C62	0.0437 (9)	0.0442 (10)	0.0355 (8)	−0.0130 (7)	−0.0176 (7)	0.0125 (7)

### Geometric parameters (Å, °)

**Table:**

O1—C2	1.4278 (18)	C41—C46	1.388 (2)
O1—C5	1.4370 (19)	C41—C42	1.397 (2)
O2—C2	1.3972 (17)	C42—C43	1.385 (2)
O2—H2O	0.8400	C42—H42	0.9500
O3—C6	1.4295 (18)	C43—C44	1.385 (3)
O3—C3	1.4254 (17)	C43—H43	0.9500
O4—C6	1.4323 (18)	C44—C45	1.386 (2)
O4—C4	1.4339 (16)	C44—H44	0.9500
C2—C3	1.522 (2)	C45—C46	1.391 (2)
C2—H2	1.0000	C45—H45	0.9500
C3—C4	1.563 (2)	C46—H46	0.9500
C3—H3	1.0000	C61—H61A	0.9800
C4—C41	1.515 (2)	C61—H61B	0.9800
C4—C5	1.535 (2)	C61—H61C	0.9800
C5—H5A	0.9900	C62—H62A	0.9800
C5—H5B	0.9900	C62—H62B	0.9800
C6—C62	1.509 (2)	C62—H62C	0.9800
C6—C61	1.513 (2)		
			
C2—O1—C5	106.30 (11)	O4—C6—C61	111.43 (13)
C2—O2—H2O	107.7	C62—C6—C61	113.45 (14)
C6—O3—C3	107.42 (11)	C46—C41—C42	118.91 (14)
C6—O4—C4	108.29 (10)	C46—C41—C4	120.93 (13)
O2—C2—O1	111.99 (12)	C42—C41—C4	120.10 (13)
O2—C2—C3	108.35 (12)	C43—C42—C41	120.58 (15)
O1—C2—C3	104.47 (11)	C43—C42—H42	119.7
O2—C2—H2	110.6	C41—C42—H42	119.7
O1—C2—H2	110.6	C44—C43—C42	120.26 (15)
C3—C2—H2	110.6	C44—C43—H43	119.9
O3—C3—C2	108.22 (12)	C42—C43—H43	119.9
O3—C3—C4	104.64 (11)	C43—C44—C45	119.46 (15)
C2—C3—C4	104.60 (11)	C43—C44—H44	120.3
O3—C3—H3	112.9	C45—C44—H44	120.3
C2—C3—H3	112.9	C46—C45—C44	120.51 (15)
C4—C3—H3	112.9	C46—C45—H45	119.7
O4—C4—C41	112.22 (11)	C44—C45—H45	119.7
O4—C4—C5	108.90 (11)	C45—C46—C41	120.27 (14)
C41—C4—C5	112.12 (12)	C45—C46—H46	119.9
O4—C4—C3	103.36 (10)	C41—C46—H46	119.9
C41—C4—C3	116.44 (11)	C6—C61—H61A	109.5
C5—C4—C3	102.99 (11)	C6—C61—H61B	109.5
O1—C5—C4	105.33 (11)	H61A—C61—H61B	109.5
O1—C5—H5A	110.7	C6—C61—H61C	109.5
C4—C5—H5A	110.7	H61A—C61—H61C	109.5
O1—C5—H5B	110.7	H61B—C61—H61C	109.5
C4—C5—H5B	110.7	C6—C62—H62A	109.5
H5A—C5—H5B	108.8	C6—C62—H62B	109.5
O3—C6—O4	104.00 (11)	H62A—C62—H62B	109.5
O3—C6—C62	109.07 (13)	C6—C62—H62C	109.5
O4—C6—C62	108.34 (12)	H62A—C62—H62C	109.5
O3—C6—C61	110.10 (13)	H62B—C62—H62C	109.5
			
C5—O1—C2—O2	−76.45 (14)	C3—O3—C6—O4	34.96 (14)
C5—O1—C2—C3	40.60 (13)	C3—O3—C6—C62	150.39 (12)
C6—O3—C3—C2	−133.84 (12)	C3—O3—C6—C61	−84.52 (14)
C6—O3—C3—C4	−22.73 (13)	C4—O4—C6—O3	−33.70 (14)
O2—C2—C3—O3	−154.79 (11)	C4—O4—C6—C62	−149.65 (13)
O1—C2—C3—O3	85.67 (13)	C4—O4—C6—C61	84.87 (14)
O2—C2—C3—C4	94.07 (13)	O4—C4—C41—C46	−14.92 (18)
O1—C2—C3—C4	−25.47 (13)	C5—C4—C41—C46	108.02 (15)
C6—O4—C4—C41	−107.03 (13)	C3—C4—C41—C46	−133.74 (14)
C6—O4—C4—C5	128.23 (12)	O4—C4—C41—C42	167.76 (12)
C6—O4—C4—C3	19.23 (13)	C5—C4—C41—C42	−69.29 (16)
O3—C3—C4—O4	2.14 (13)	C3—C4—C41—C42	48.95 (18)
C2—C3—C4—O4	115.84 (12)	C46—C41—C42—C43	0.4 (2)
O3—C3—C4—C41	125.66 (12)	C4—C41—C42—C43	177.73 (13)
C2—C3—C4—C41	−120.64 (13)	C41—C42—C43—C44	−0.2 (2)
O3—C3—C4—C5	−111.22 (12)	C42—C43—C44—C45	0.2 (2)
C2—C3—C4—C5	2.48 (13)	C43—C44—C45—C46	−0.4 (2)
C2—O1—C5—C4	−39.18 (13)	C44—C45—C46—C41	0.6 (2)
O4—C4—C5—O1	−88.03 (13)	C42—C41—C46—C45	−0.6 (2)
C41—C4—C5—O1	147.17 (11)	C4—C41—C46—C45	−177.92 (13)
C3—C4—C5—O1	21.22 (13)		

### Hydrogen-bond geometry (Å, °)

**Table:**

*D*—H···*A*	*D*—H	H···*A*	*D*···*A*	*D*—H···*A*
O2—H2o···O1^i^	0.84	1.93	2.755 (2)	166
C5—H5a···O2^ii^	0.99	2.47	3.296 (3)	140

Symmetry codes: (i) −*x*, −*y*+2, −*z*+1; (ii) *x*−1, *y*, *z*.
